# DEVELOPING A FITNESS AGE-NORMALIZED PERCENTILE-BASED APPROACH TO DEFINE EXCEPTIONAL AGING

**DOI:** 10.1093/geroni/igac059.1235

**Published:** 2022-12-20

**Authors:** Eleanor Simonsick, Ann Moore, Toshiko Tanaka, Qu Tian, Luigi Ferrucci

**Affiliations:** National Institute on Aging, Baltimore, Maryland, United States; TGB NIA NIH, Baltimore, Maryland, United States; National Institute on Aging, Baltimore, Maryland, United States; National Institutes on Aging, Baltimore, Maryland, United States; National Institute on Aging, Baltimore, Maryland, United States

## Abstract

An operational definition of ‘well-aged’ is integral to research identifying biological, psychological and behavioral parameters that predict and/or support healthy aging but remarkably few measures exist. A major challenge is incorporating age-related physiologic decline – a normal feature of healthy aging. We defined well-aged using fast 400m walk performance, a measure of cardiovascular fitness and hallmark of healthy aging. We determined the sex-specific, age-normalized 75th percentile using 4000+ observations collected over 15 years from BLSA participants aged 50-96y (51% women). In 1257 BLSA participants mean age 68.9y (52% women) we compared well-aged to average-aged (25th-75th percentile) adjusting for age, sex and race, and found more favorable WBC, triglycerides, CRP, depressive symptoms, waist/height ratio, eGFR and FEV1 z-score distinguished concurrent well-aged status (all p<.003). Waist/height ratio and pulse-wave velocity (both p<.001) predicted retained well-aged status 2-plus years later. Future work aims to identify physiologic and behavioral facilitators of healthy aging.

